# Estimation of missing data of monthly rainfall in southwestern Colombia using artificial neural networks

**DOI:** 10.1016/j.dib.2019.104517

**Published:** 2019-09-14

**Authors:** Teresita Canchala-Nastar, Yesid Carvajal-Escobar, Wilfredo Alfonso-Morales, Wilmar Loaiza Cerón, Eduardo Caicedo

**Affiliations:** a Grupo de Investigación en Ingeniería de Recursos Hídricos y Suelos (IREHISA), Escuela de Recursos Naturales y del Ambiente (EIDENAR), Facultad de Ingeniería, Universidad del Valle, Calle 13 # 100-00, Cali, Colombia; b Grupo de Percepción y Sistemas Inteligentes (PSI), Escuela de Ingeniería Eléctrica y Electrónica, Facultad de Ingeniería, Universidad del Valle, Calle 13 # 100-00, Cali, Colombia; c Departamento de Geografía, Facultad de Humanidades, Universidad del Valle, Calle 13 # 100-00, Cali, Colombia

**Keywords:** Missing data, Monthly Rainfall Data, Artificial neural networks, NLPCA

## Abstract

The success of many projects linked to the management and planning of water resources depends mainly on the quality of the climatic and hydrological data that is provided. Nevertheless, the missing data are frequently found in hydroclimatic variables due to measuring instrument failures, observation recording errors, meteorological extremes, and the challenges associated with accessing measurement areas. Hence, it is necessary to apply an appropriate fill of missing data before any analysis. This paper is intended to present the filling of missing data of monthly rainfall of 45 gauge stations located in southwestern Colombia. The series analyzed covers 34 years of observations between 1983 and 2016, available from the *Instituto de Hidrología, Meteorología y Estudios Ambientales (IDEAM).* The estimation of missing data was done using Non-linear Principal Component Analysis (NLPCA); a non-linear generalization of the standard Principal Component Analysis Method via an Artificial Neural Networks (ANN) approach. The best result was obtained using a network with a [45−44−45] architecture. The estimated mean squared error in the imputation of missing data was approximately 9.8 mm. month^−1^, showing that the NLPCA approach constitutes a powerful methodology in the imputation of missing rainfall data. The estimated rainfall dataset helps reduce uncertainty for further studies related to homogeneity analyses, conglomerates, trends, multivariate statistics and meteorological forecasts in regions with information deficits such as southwestern Colombia.

**Table:** Specifications Table

Subject	Environmental Science and Artificial Intelligence
More specific subject area	Application of neural networks in the estimation of missing data in rainfall time series
Type of data	Figures and tables
How data was acquired	Rainfall data were obtained following the formal application procedure of the IDEAM (Colombia).
Data format	Raw and Analyzed data
Experimental Factors	Monthly rainfall
Experimental Features	Estimation missing rainfall data through NLPCA, an auto-associative neural network, generally seen as a non-linear generalization of standard linear principal component analysis.
Data source location	Southwestern Colombia (Nariño)
Data accessibility	Data are available in this article

**Table:** 

**Value of the data** • The data in this article can be used to: a) enhance the consistency of water-resources management studies, b) improve the trend analyses of rainfall, c) identify homogeneous climatic regions and d) diminish the uncertainty of the rainfall forecast. • The dataset is useful for the institutions, researchers, and experts that are involved in the management of water resources, risk management, food safety and other fields linked to climatic variability. • The estimated rainfall data can be used as input of forecast models, statistical models, and different linear and non-linear techniques for the analysis of the rainfall variability. • The estimated dataset provides new rainfall information in one of the most biodiverse regions in the country and the rest of the world.

## 1 Data

**Fig. 1:**
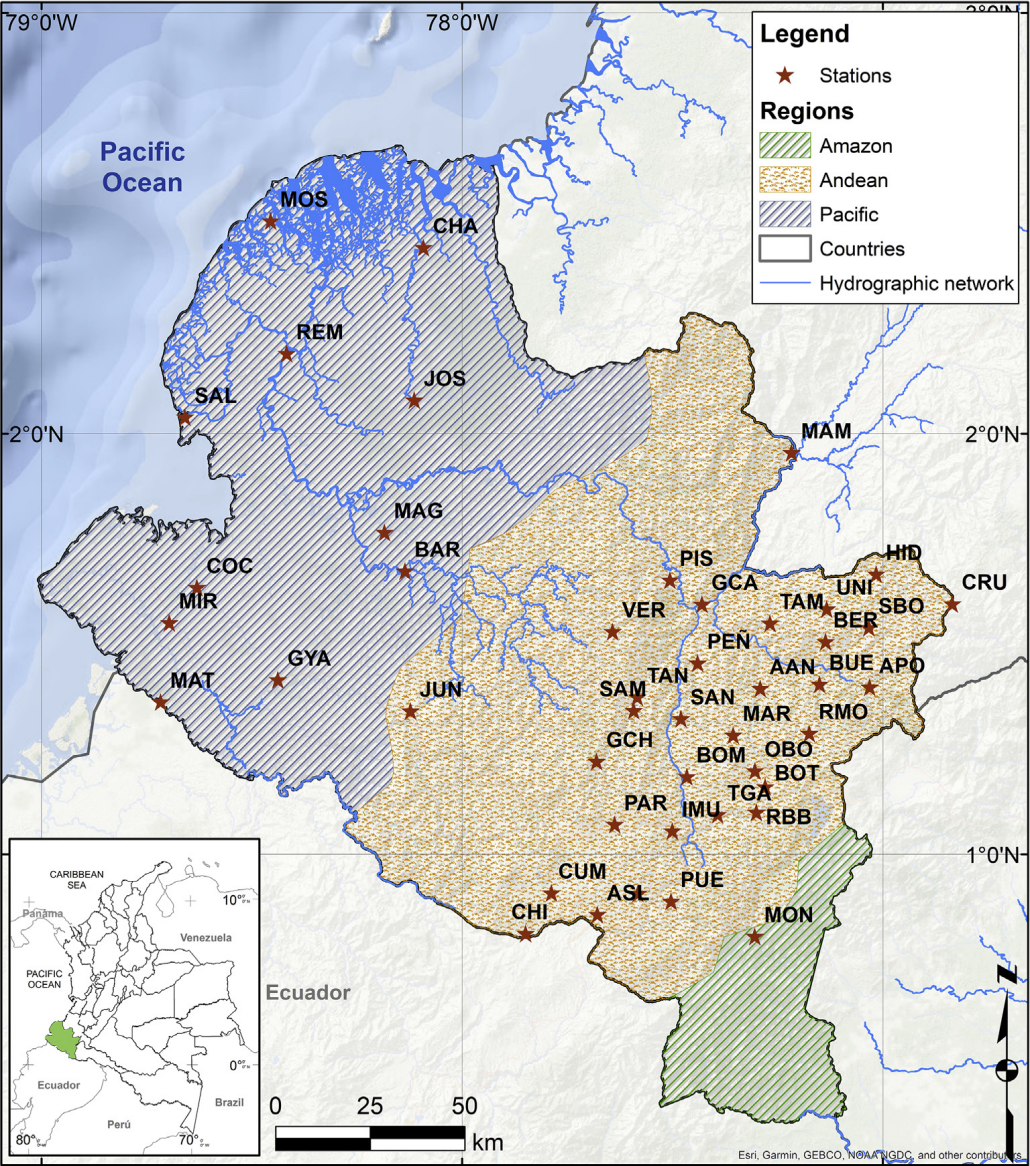
Location of rainfall gauge stations in Nariño (southwestern Colombia).

**Fig. 2:**
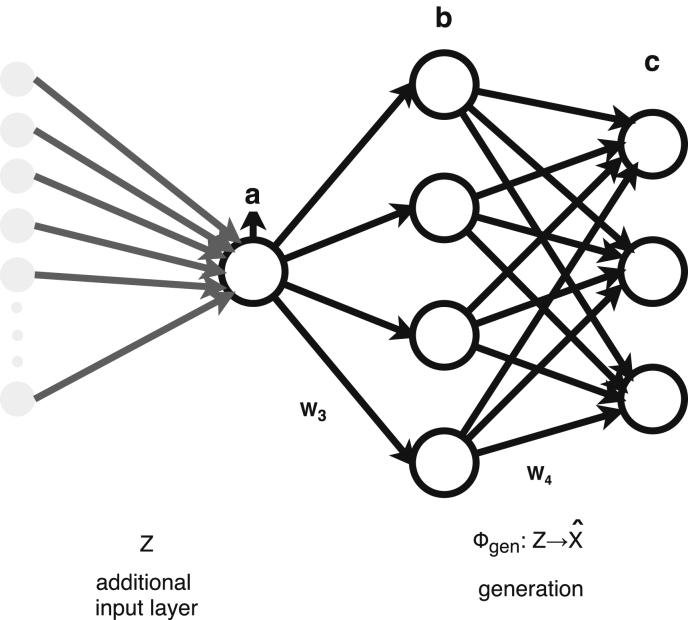
A schematic diagram of the inverse NLPCA model. Network with a [*a-b-c*] architecture.

**Fig. 3:**
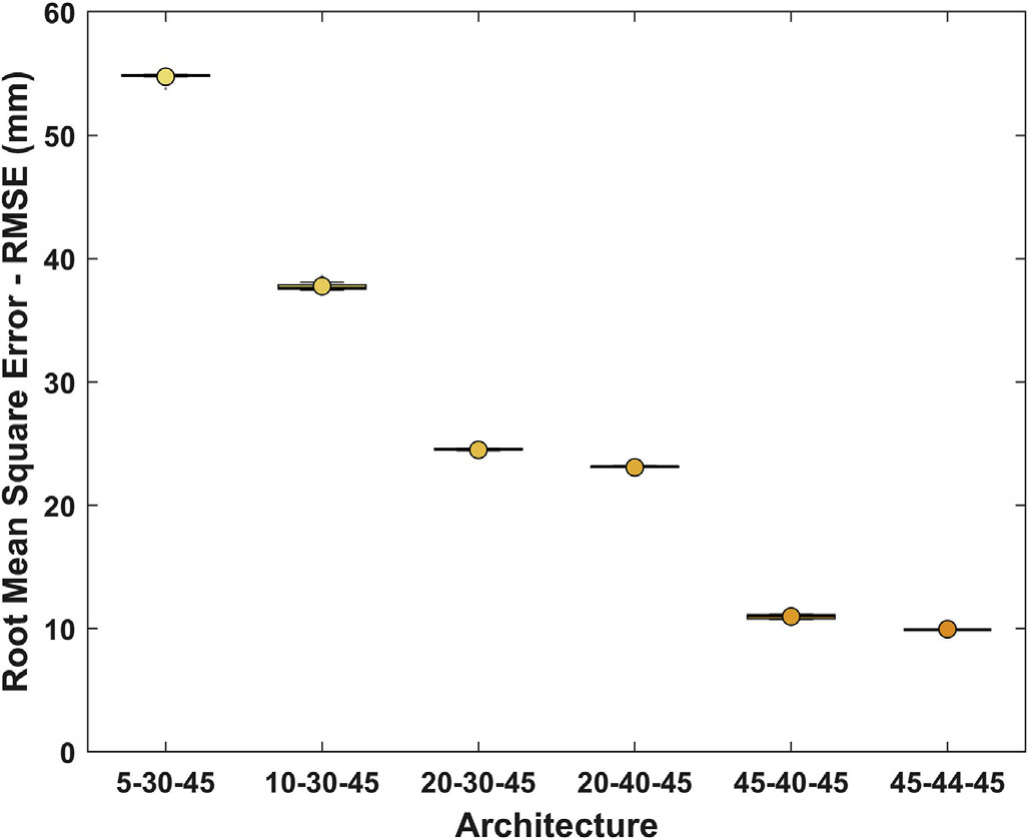
Architectures used for the selection of the best ANN.

**Fig. 4:**
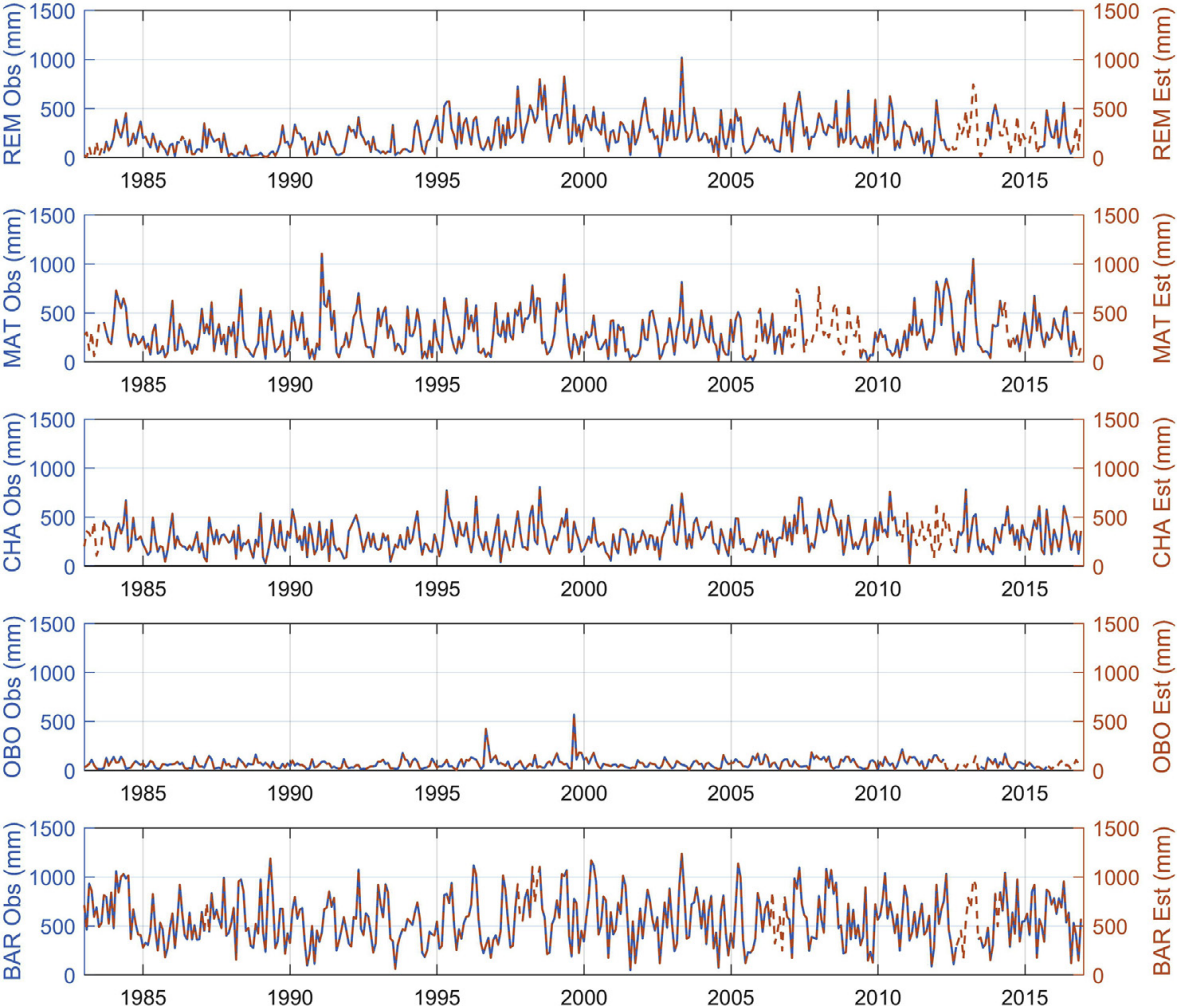
Time series observed rainfall vs estimated rainfall of REM, MAT, CHA, OBO, and BAR rainfall stations.

**Fig. 5:**
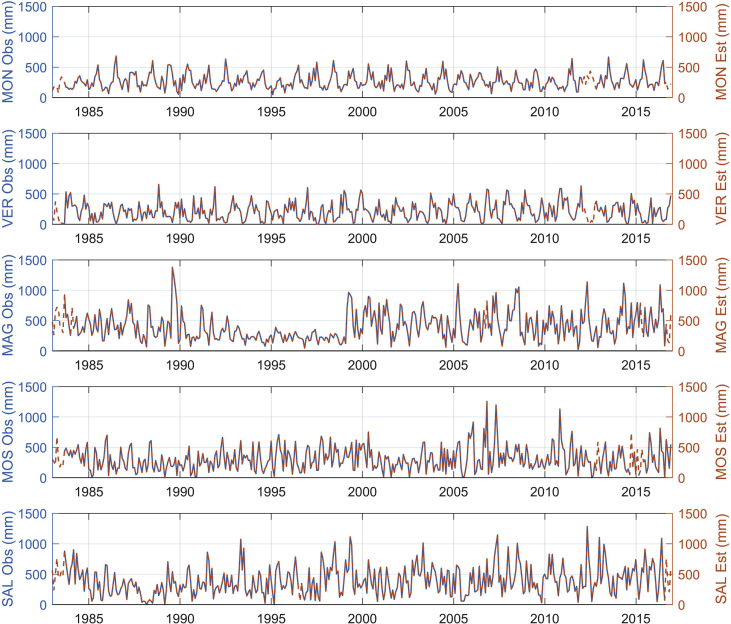
Time series observed rainfall vs estimated rainfall of MON, VER, MAG, MOS, and SAL rainfall stations.

**Fig. 6:**
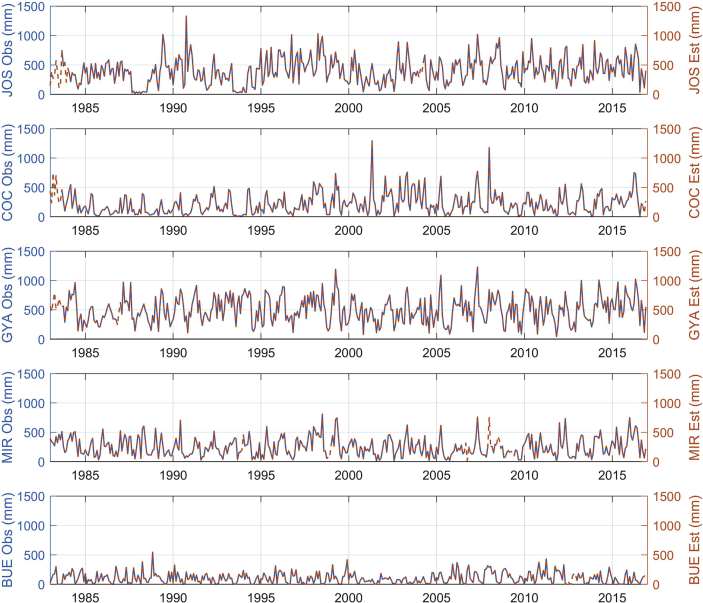
Time series observed rainfall vs estimated rainfall of JOS, COC, GYA, MIR, and BUE rainfall stations.

The figures and tables of monthly rainfall were analyzed based on the data obtained from 45 stations located in different zones in the Department of Nariño (Colombia). Fig. 1 is the location map of rainfall stations. Descriptive statistical analysis of the monthly rainfall data (1983–2016) is presented in Table 1. Fig. 2 shows the schematic diagram of the inverse Non-Linear Principal Components Analysis (NLPCA) model. Fig. 3 shows Artificial Neural Network (ANN) modelling data for ten trainings per architecture. The best estimates from the different architectures with the inverse NLPCA model are shown in Table 2. The time series of rainfall stations with the percentage of missing data higher than 1% in the original data and the estimation of missing data are divided and presented in three graphs in descending order in Fig. 4, Fig. 5, and Fig. 6, so that they are easier to observe. With this in mind: Fig. 4 shows the time series for REM, MAT, CHA, OBO, and BAR (see ID and Missing Data Percent in Table 1); Fig. 5 shows the time series for MON, VER, MAG, MOS, and SAL; and lastly, Fig. 6 presents the time series for JOS, COC, GYA, MIR, and BUE.

**Table 1:** Descriptive statistical analysis of the monthly rainfall data in Nariño (1983–2016).

Station	ID	Rainfall mean (mm.year^−1^)	Rainfall Std div (mm.year^−1^)	Rainfall CV	Missing Data %
Barbacoas	BAR	562.8	256.4	0.46	4.41
Berruecos	BER	145.4	110.8	0.76	0.74
Bombona	BOM	86.3	61.1	0.71	0.25
Botana	BOT	76.3	40.2	0.53	0.74
Buesaco	BUE	104.6	92.1	0.88	1.23
Chiles	CHI	91.4	61.6	0.67	0.98
Cumbal	CUM	75.2	47.8	0.64	0.00
Paraiso	PAR	82.4	52.4	0.64	0.49
Peñol	PEÑ	90.4	65.4	0.72	0.00
Mira	MIR	250.0	159.5	0.64	2.21
Guachavez	GCH	138.1	91.7	0.66	0.74
Gualmatán	GMT	77.5	53.7	0.69	0.74
Hidromayo	HID	110.3	88.3	0.80	0.49
Imues	IMU	83.5	60.3	0.72	0.25
Junin	JUN	726.6	270.7	0.37	0.49
Charco	CHA	301.6	148.2	0.49	6.86
Guasca	GCA	49.0	46.5	0.95	0.00
La Unión	UNI	164.6	115.4	0.70	0.49
Mamaconde	MAM	107.5	93.8	0.87	0.98
Mataje	MAT	289.7	197.1	0.68	9.07
Mosquera	MOS	304.4	198.4	0.65	3.19
Nariño	NAR	165.5	126.0	0.76	0.00
Obonuco	OBO	68.7	52.4	0.76	6.62
Pisanda	PIS	105.6	79.7	0.75	0.00
Puerres	PUE	84.9	49.1	0.58	0.00
Remolino	REM	228.6	163.4	0.72	10.78
Rio Bobo	RBB	91.7	53.4	0.58	0.98
Rosal Monte	RMO	111.9	90.1	0.81	0.98
Salahonda	SAL	396.5	238.2	0.60	3.19
Samaniego	SAM	122.1	98.4	0.81	0.00
San Bernardo	SBO	167.0	116.7	0.70	0.49
Jose Tapaje	JOS	399.8	222.4	0.56	2.70
Sandoná	SAN	95.2	78.4	0.82	0.98
Taminango	TAM	140.9	95.8	0.68	0.98
Tanama	TAN	112.9	80.3	0.71	0.98
Tangua	TGA	83.8	60.8	0.73	0.49
La cruz	CRU	112.0	91.5	0.82	0.98
Monopamba	MON	267.8	125.6	0.47	4.41
A. San Luis	ASL	72.5	43.4	0.60	0.00
A. Antonio Nariño	AAN	98.1	71.5	0.73	0.00
Aponte	APO	128.6	116.1	0.90	0.00
Vergel	VER	214.8	145.1	0.68	3.68
Magui	MAG	404.4	236.5	0.58	3.43
Guayacana	GYA	500.2	223.6	0.45	2.21
Coco	COC	215.5	180.2	0.84	2.45

**Table 2:** Data of root-mean-square-error (RMSE) for different architecture evaluated.

Architecture	Explained Variance Components (%)	RMSE (mm)
5–30–45	80.9	±53.7
10–30–45	93.4	±37.5
20–30–45	96.1	±24.5
20–40–45	96.4	±23.2
45–40–45	98.0	±10.9
45–44–45	98.6	±9.8

## 2 Experimental design, materials and methods

### 2.1 Study area description

The Department of Nariño, located in southwestern Colombia, covers an area of 33,268 km^2,^ with a geo-strategic position between the Colombian-Ecuadorian border, the tropical Pacific Ocean and the Andes mountain range of South America. 8% of its territory belongs to the Amazon basin, one of the largest biodiversity reserves in the world; 52% corresponds to the Chocó biogeographic plains that harbor a mega-diversity of species; and the remaining 40% covers the Andean region, where paramos and volcanoes dominate [1]. These are aspects that make Nariño one of the most diverse regions of Colombia, and the rest of the world (Fig. 1).

### 2.2 Material and methods

The imputation of missing rainfall data was performed through the inverse model of the NLPCA technique defined by Scholz et al. [2], [3] and applied to the estimation of missing data of rainfall by Miró et al. [4], who concluded that the NLPCA technique presented the best results among the ten methods implemented for data estimation. Consequently, while classical ANN forward approach is driven by two steps: i) Extraction function ${\text{Φ}}_{extr}:X\to Z$ and ii) Reconstruction function ${\text{Φ}}_{gen}:Z\to \hat{X}$ , the inverse NLPCA is only driven by the second part [2]. Inverse NLPCA (Fig. 2) is led by the mapping function ${\text{Φ}}_{gen}$, which is performed by a feed-forward network. Eq. (1) shows the output $\hat{X}$ is dependent upon the input Z and the ANN weights $w\varepsilon W_{3},W_{4}$.

(1) $$\hat{X}={\text{Φ}}_{gen}\left(w,Z\right)=W_{4}g\;\left(W_{3}Z\right)$$

The goal is to achieve a function ${\text{Φ}}_{gen}$ capable of generating data $\hat{X}$ that approximates the target data X by minimizing the squared error $X-{\hat{X}}^{2}$. Biases are not explicitly considered; however, they can be included by introducing an extra unit, or input, with activation ﬁxed at one. The architecture of inverse NLPCA model is [*a-b-c*], where *a* are the extracted non-linear components, *b* are the non-linear hidden units used to perform the non-linear transformation and *c* are the approximated features. A further explanation and details about this process are available in Scholz et al. [2], [3].

The inverse NLPCA is not limited to one component; it can be extended to *m* components with an additional hierarchically error function [5]. The non-linear components 1,…,m can be extracted in a hierarchical order, which is a natural non-linear extension to the hierarchical ordered components of the standard linear PCA. For the application of NLPCA, the Nonlinear PCA toolbox (available in http://www.nlpca.org/matlab.html) was used. Here, the hierarchical NLPCA was used to get the hierarchically ordered features by training sequentially, where the remaining variance/error allows to calculate the explained variance, despite of it cannot be considered regardless of the nonlinear mapping [5]. Regarding the application of the inverse NLPCA, different architectures were tested (Table 2), and an increase in the explained variance and a decrease in the Root Mean Square Error (RMSE) was observed when the number of non-linear principal components was increased. The best estimates were obtained using a network with a [45−44−45] architecture (Fig. 3). This means we have extracted 45 non-linear components; 44 non-linear hidden units were used to perform the non-linear transformation, and 45 rainfall gauge stations were approximated. The parameters setting that presented a better result was: weight decay coefficient set at 0.01 and the maximum number of iterations set at 5,000. The components were extracted in a hierarchical order. All other parameters were set by default (type inverse, no circular PCA). The final imputation results can be different for each model obtained by the inherent characteristics of ANN. Hence, for each execution, the NLPCA was trained ten times, choosing as a priori the one with the best performance in terms of RMSE.

## References

[1] Gobernación de Nariño, Plan participativo de desarrollo departamental 2016-2019. http://nariño.gov.co/inicio/index.php/gobernacion/plan-de-desarrollo-departamental-narino-corazon-del-mundo-2016-2019 (accesed 21 August 2019).

[2] Scholz M., Kaplan F., Guy C.L., Kopka J., Selbig J. (2005). Non-linear PCA: a missing data approach. Bioinformatics.

[3] Scholz M., Fraunholz M., Selbig J. (2008). Nonlinear principal component analysis: neural network models and applications. Principal Manifolds for Data Visualization and Dimension Reduction.

[4] Miró J.J., Caselles V., Estrela M.J. (2017). Multiple imputation of rainfall missing data in the Iberian Mediterranean context. Atmos. Res..

[5] Scholz M., Vigário R. (2002). Nonlinear PCA: a new hierarchical approach. ESANN.

